# Health system integration with physician specialties varies across markets and system types

**DOI:** 10.1111/1475-6773.13584

**Published:** 2020-12-07

**Authors:** Rachel M. Machta, James D. Reschovsky, David J. Jones, Laura Kimmey, Michael F. Furukawa, Eugene C. Rich

**Affiliations:** ^1^ Mathematica Oakland California USA; ^2^ JR Health Policy Consultants, LLC Rockville Maryland USA; ^3^ Mathematica Cambridge Massachusetts USA; ^4^ Mathematica Washington District of Columbia USA; ^5^ Agency for Healthcare Research and Quality Rockville Maryland USA

**Keywords:** Healthcare Organizations and Systems, integrated delivery systems, ownership governance

## Abstract

**Objective:**

To examine system integration with physician specialties across markets and the association between local system characteristics and their patterns of physician integration.

**Data Sources:**

Data come from the AHRQ Compendium of US Health Systems and IQVIA OneKey database.

**Study Design:**

We examined the change from 2016 to 2018 in the percentage of physicians in systems, focusing on primary care and the 10 most numerous nonhospital‐based specialties across the 382 metropolitan statistical areas (MSAs) in the US. We also categorized systems by ownership, mission, and payment program participation and examined how those characteristics were related to their patterns of physician integration in 2018.

**Data Collection/Extraction Methods:**

We examined local healthcare markets (MSAs) and the hospitals and physicians that are part of integrated systems that operate in these markets. We characterized markets by hospital and insurer concentration and systems by type of ownership and by whether they have an academic medical center (AMC), a 340B hospital, or accountable care organization.

**Principal Findings:**

Between 2016 and 2018, system participation increased for primary care and the 10 other physician specialties we examined. In 2018, physicians in specialties associated with lucrative hospital services were the most commonly integrated with systems including hematology‐oncology (57%), cardiology (55%), and general surgery (44%); however, rates varied substantially across markets. For most specialties, high market concentration by insurers and hospital‐systems was associated with lower rates of physician integration. In addition, systems with AMCs and publicly owned systems more commonly affiliated with specialties unrelated to the physicians’ potential contribution to hospital revenue, and investor‐owned systems demonstrated more limited physician integration.

**Conclusions:**

Variation in physician integration across markets and system characteristics reflects physician and systems’ motivations. These integration strategies are associated with the financial interests of systems and other strategic goals (eg, medical education, and serving low‐income populations).

**What is known on the topic**

- Physician integration with health systems varies substantially by region and market size, and there is some evidence that it is more common for specialties lucrative to hospitals, especially cardiology and oncology.
- Physician‐system integration has led to higher negotiated prices with private insurers, with little or no improvements in quality of care, but the integration examined in these studies does not account for differences in integration across physician specialties and diverse systems and markets.
- There is still a lot that is not known about how markets and health system characteristics are related to the levels of physician integration across specialties, which could help inform future studies on the impacts of integration on key outcomes.

**What this study adds**

- Between 2016 and 2018, system participation increased for primary care and the 10 other physician specialties we examined; however, it was greatest for specialties lucrative to hospitals and uneven across systems and markets.
- In most cases, high market concentration by insurers and hospital‐systems were associated with lower rates of physician integration, and publicly owned systems and systems with an academic medical center integrated with a broader set of physician specialties compared to investor‐owned systems.
- Policymakers considering actions to prevent or address potentially negative consequences of physician integration with systems should consider the context of local markets and local health systems as well as how payment policies might encourage integration with physician specialties that enhances value.

## 1 INTRODUCTION

Vertically integrated health systems have become increasingly prominent in the US health care delivery system (defined in this paper as hospitals and physicians with common ownership or joint management).^1^ Between 2016 and 2018, physician integration with systems increased substantially (from 40% to 51% of physicians), while hospital integration with systems increased little (from 70% to 72% of hospitals).^2^

Previous studies have shown physician integration with systems varies considerably by region and market size and grew more rapidly among physician specialties lucrative to hospitals, especially cardiology and oncology.^3^ Physician integration in 2018 was higher in the Northeast and Midwest than the South and West, and the markets with the largest increases in physician integration from 2016 to 2018 were relatively small.^4^

Integration of physicians with systems is of key policy importance. Multiple studies have shown that physician‐system integration has led to higher negotiated prices with private insurers, with little or no improvements in quality of care.^5^, ^6^, ^7^, ^8^ However, these aggregate findings may obscure important differences that emerge as diverse types of systems implement varying physician integration strategies. These strategies may be reflected by the degree to which the systems rely on integrated physicians versus independent physicians for inpatient and outpatient services and by the mix of physicians across specialties that are integrated with the systems. The purpose of this study is to examine changes in physician integration from 2016 to 2018, overall and by physician specialties, and to explore how integration varies by market and system characteristics in 2018.

### 1.1 Framework for vertical integration of hospitals and physicians

A key financial motivation for systems to integrate with physicians is to maintain or grow the system's share of lucrative inpatient and outpatient services by ensuring these services are performed within the system. As a result of changes in technology, clinical practice, and payment policies, many procedures that have historically been provided in hospital facilities (in either inpatient or hospital outpatient department sites), are now provided in other outpatient settings (eg, coronary angiography). Moreover, other services, like advanced imaging, are now commonly provided outside hospital outpatient departments in physician offices, imaging centers, and other settings. System integration with physicians provides an opportunity for local systems to defend or grow their market share for these services, neutralize niche competitors, and preempt new market entry.^9^

Systems may also integrate with specialist and primary care physicians to gain patient referrals for hospital admissions, outpatient department services, or visits to system‐integrated specialists.^10^ Independent physicians might refer patients to multiple hospitals or physician specialists, but systems have more effective means to facilitate integrated physicians referring to system providers.^11^, ^12^, ^13^ Some forms of physician incentives may be constrained by anti‐kickback statutes (eg, the Stark Law) but systems may still find ways to encourage providers to alter treatment patterns to encourage greater use of lucrative services.^11^

System opportunities to charge higher prices may also provide financial motivations for hospital‐physician integration. Studies have shown that system providers are associated with higher prices, in part due to greater leverage in negotiations with insurers resulting from their hospital and physician market share as well as from the ability to negotiate jointly for physicians and hospitals.^5^, ^6^, ^7^, ^8^, ^13^, ^14^ In addition, integration enables systems to capture hospital facility fees by billing services provided in offices of employed physicians as taking place in hospital outpatient departments.^7^

Systems may also integrate with physicians to provide greater levels of clinical integration and efficiency. Clinical integration can occur through better information sharing, communication, and coordination between hospitals, physicians, and other system providers and may result in improved care transitions and quality. Clinical integration in turn can lead to greater efficiency through reductions in unnecessary care and medical errors and improved adherence to evidence‐based practices.^9^ A key impetus for achieving these efficiencies comes from patient safety initiatives as well as alternative payment models (APMs) that reward improved quality and lower costs. Even if a system is not heavily engaged in APMs, it may wish to position itself to succeed under such models, should policies change, or market dynamics compel them.^10^ Integrated systems that largely function as insurers (eg, Kaiser Permanente) might prioritize clinical integration and efficiency while deemphasizing the financial considerations described above.

Beyond the above mentioned financial or clinical motivations for systems to integrate with physicians, the functions and goals of specific health systems vary, likely leading to differing physician integration strategies. Systems with different types of ownership (eg, public or investor) may have varying missions that are manifested in part through differing approaches to physician integration.^15^ For example, systems with public ownership might aim to provide access to essential services to disadvantaged populations by integrating providers who offer those services into their system.

Prominent motivations for physicians to join systems include stagnant or declining reimbursements, the rising burdens of running an independent practice, and a desire for better work‐life balance.^10^ In part, growing burdens are the result of increased practice requirements that accompany health care payment reforms, including federal incentives for meaningful use of electronic health records and the complex and evolving requirements for participation in APMs.^16^ Integration with systems relieves physicians of direct responsibility for these requirements and typically shares the costs of complying with these regulations across a larger number of providers.

Physician training emphasizes personal responsibility for high‐quality patient care.^17^ While integration with systems may threaten professional autonomy, this challenge may be offset by increased access to up‐to‐date facilities and technology conferred through systems’ greater access to capital. However, physicians may derive similar benefits, with perhaps fewer restrictions on clinical autonomy, via integration with large physician organizations. The local availability of these alternatives can vary and is likely associated with variation in physician integration with systems across markets.^18^ Based on the above considerations, we offer the following expectations.

### 1.2 Expectation 1

Physicians in specialties that provide lucrative inpatient and outpatient hospital services (such as cardiologists, oncologists, orthopedists, and general surgeons) are likely to be integrated with systems at higher rates than physicians that seldom use hospital services. This integration is due to financial motivations for systems to protect and grow hospital revenue, much of which falls in service lines associated with these specialties. However, there is likely greater variation in physician integration across markets in these specialties relative to others due to the ability of physicians in these specialties to capture revenue independent of hospitals and systems (eg, through ownership of ambulatory surgical centers or market power achieved through horizontal integration into large practices).

### 1.3 Expectation 2

The market concentration in both hospital and insurer markets will be associated with different levels of physician integration as a result of differing opportunities to negotiate higher prices.^19^, ^20^, ^21^, ^22^, ^23^ Though these relationships are complex and possibly related, we generally expect that integration will have an inverse relationship with concentration of both hospital and insurer markets. Systems in more concentrated (ie, less competitive) hospital markets that already have considerable control over the inpatient and outpatient hospital market may find fewer marginal benefits from acquiring additional physicians to solidify their market position. As a result, these markets will generally be associated with less physician‐system integration. We expect this trend will also be true for insurance markets. That is, more concentrated (less competitive) health insurance markets will generally have less physician‐system integration because the upside of employing physicians to gain leverage in negotiations with payers will be diminished.

### 1.4 Expectation 3

The ownership and mission of systems will be associated with their physician integration strategies. Public systems centered around a safety net hospital may view providing access to care for disadvantaged populations as a key organizational mission.^24^ As a result, such systems may emphasize physician integration strategies focused on important specialties its patients have difficulty accessing, such as ophthalmology and psychiatry, even if those specialties do not support lucrative hospital service lines. Similarly, systems that include an academic medical center (AMC) are likely to integrate with physicians to fulfill their teaching and research goals such as ensuring teaching sites for students, securing faculty for advanced training programs, or accessing patients for priority research initiatives. Therefore, such systems may integrate with physicians across a broader range of specialties, including those that seldom generate referrals for inpatient or lucrative hospital outpatient services (eg, dermatologists).

### 1.5 Expectation 4

Systems will alter their physician integration patterns to maximize revenue under APMs or other payment programs. Health systems in payment models that reward accountability for total cost of care for a population (such as Accountable Care Organizations [ACOs]) will be more likely to integrate with primary care physicians and physicians in specialties focused on prevalent chronic conditions (eg, gastroenterology, ophthalmology, cardiology, and hematology‐oncology). In addition, the 340B drug discount program enables qualifying hospitals to purchase drugs at steep discounts for patients that receive care from their affiliated physicians, but they need not pass these savings on to patients using these drugs. This feature of the program could provide systems that include 340B hospitals an incentive to integrate with physicians in specialties, such as hematologists‐oncologists, that prescribe large quantities of expensive drugs.^25^

## 2 DATA AND METHODS

To address our hypotheses, we examined healthcare markets and the local health systems that operate within these markets. At each level, we explored the extent of physician integration overall and across physician specialties. We define healthcare markets as metropolitan statistical areas (MSAs). We define local health systems as the hospitals and physicians that are owned or managed by the same system in an MSA. Systems that operate in multiple markets have multiple records, one for each market in which they operate.

We identified systems using the 2016 and 2018 AHRQ Compendium of US Health Systems, a publicly available database with information on all systems operating in the United States.^26^ AHRQ used a consensus process among a broad range of national experts to develop a definition of a health system. A system is defined to include at least one hospital and at least one group of physicians that provide comprehensive services who are connected through common ownership or joint management. Specifically, Compendium systems must have at least one nonfederal general acute care hospital and 50 or more physicians, including 10 or more primary care physicians nationally (regardless of whether they are organized as a separate medical group).^26^

We use local health systems because many Compendium systems operate in more than one market (sometimes operating nationally). Each market a system operates in is unique, and therefore, we expect that systems’ physician integration strategies will be tailored to specific markets. We defined local health systems as the Compendium systems with at least one general acute care hospital and at least one physician in the MSA with common ownership or joint management. Therefore, our definition excludes market operations of Compendium systems that do not include a general acute care hospital (eg, Kaiser Permanente outside of the West Coast and Hawaii) or do not include any physicians within an MSA.

We identified physicians and system affiliation (if any) using the 2016 IMS Healthcare Organization Services (HCOS) data, and for 2018, its successor, the IQVIA OneKey data. We used the same methodology as the Compendium to link physicians to the 2016 and 2018 versions of the Compendium. Specifically, we consider physicians to be affiliated with system if the data indicate tight affiliations with a facility in the system; we did not consider physicians with loose affiliations such as admitting privileges at hospitals to be system‐owned.^26^ A small number of physicians appear in multiple markets or multiple local systems. In our market‐level analysis, we limited each physician to no more than one record per MSA.

We focused on the 10 nonhospital‐based specialties with the largest number of active physicians according to the Association of American Medical Colleges (AAMC): obstetrics‐gynecology, psychiatry, general surgery, cardiology, orthopedic surgery, ophthalmology, gastroenterology, hematology‐oncology, neurology, and dermatology.^27^ Additionally, we included a primary care category, comprising adolescent medicine, family medicine, geriatrics, general practice, internal medicine, and pediatrics. In the Appendix S1, we report physician counts from OneKey, HCOS, and AAMC data, by specialty.

We begin by examining physician integration at the market level. There are 382 MSAs across the US. We use our market‐level file to characterize physician integration across markets and examine whether integration is associated with hospital‐system and insurance market concentration. We then delve into the local systems operating in those markets and examine local system characteristics that are associated with different physician integration strategies. We identified 1158 local health systems operating across 373 MSAs in 2018 (9 MSAs had no local health systems meeting our definition).

### 2.1 Market‐level measures

We calculated the market‐level degree of system integration as the percentage of all physicians in the market who were integrated with systems in 2016 and in 2018. We report this measure for all physicians and by physician specialty.

We used the Herfindahl‐Hirschman Index (HHI) to measure market concentration of hospital services and health insurance in 2018. HHI is calculated as the sum of the squared market shares of all “firms” in a market. Higher HHIs indicate greater concentration. In calculating the hospital‐system HHI, we defined firms as either single nonsystem hospitals or as all hospitals in the MSA associated with a system. We defined market share in terms of the total number of hospital inpatient discharges (using data from the Medicare Healthcare Cost Report Information System [HCRIS]). Although we use inpatient discharges to calculate market concentration in hospital services, levels of outpatient services are likely similar, and inclusion of outpatient services is unlikely to change the categorization of MSAs by HHI given the broad categories we use to group MSAs (see below).

We used data from the American Medical Association on health insurance HHI. This measure captures concentration for commercial insurance products (including PPO, HMO, POS, and public exchange enrollment) and is reported at the MSA level.^28^ We used HHI values to identify whether MSAs have high or low concentration based on hospitals and insurers. Hospital‐system and insurer concentration HHI ranged from 472 to 10 000 (median 5909) and 1518 to 8344 (median 3211), respectively. The Department of Justice and Federal Trade Commission Horizontal Merger Guidelines categorizes markets with a HHI above 2500 as highly concentrated.^29^ Because the MSA‐level HHI values are skewed toward higher values (91% and 75% of MSAs are above the cutoff for highly concentrated hospital and insurer markets, respectively), we use terciles rather than this single cutoff to define cut points. We classified markets as having relatively low concentration (more competitive) if they were in the lowest tercile across markets: HHIs less than or equal to 4696 for hospital‐system concentration and 2696 for insurer concentration. We classified markets as having relatively high concentration (less competitive) if they were in the highest tercile: HHIs greater or equal to 6886 for hospital concentration and 3780 for insurer concentration.^28^

We examined the association between market concentration and the percentage of physicians overall and by specialty in the market who were integrated with systems in 2018. Because concentration and integration measures might vary by market size, we used linear regression to adjust physician integration measures for total population in the MSA.

### 2.2 Local health system‐level measures

We created indicator variables for local health system integration with specific physician specialties based on whether they had one or more physicians in the market for each specialty we examined. In addition, we created a count of specialties included in the local system as a simple way to summarize the variation across systems in integration with physician specialties. This count can range from 1 to 11 and is the sum of the indicators described above.

We also created a measure of the degree to which systems integrate with a physician specialty relative to the system's hospital capacity. To do so, we constructed a vertical integration scale defined as the number of physicians in the local system (overall, and by specialty) divided by the number of beds in the local system. This metric provides a rough measure for the degree of care provided by a local system that might be provided by integrated physicians. Because this measure's absolute values are not easily interpretable and vary with the relative prevalence of each physician specialty (and that specialty's relevance to hospital beds), we normalized values by the within physician specialty mean across all local systems. Therefore, for each specialty, the measure reflects a local system's degree of vertical integration relative to the national average of all local systems. A value above one indicates relative integration with physicians above the national average for that specialty.

### 2.3 Local health system characteristics

We examined integration by local system characteristics that typify differing missions and payment opportunities. We categorized health system ownership as either public, not‐for‐profit, or investor‐owned based on plurality ownership of system hospitals (weighted by hospital beds) reported in the HCRIS data. We also identified local systems that included an AMC. A list of AMCs and their parent systems in 2016 came from a study of academically affiliated health systems.^30^ That study defined AMCs as hospitals with a resident‐to‐bed ratio of at least 0.25 that were affiliated with at least one medical school. We created a variable indicating whether the local system included at least one hospital participating in an ACO using data from the 2016 Leavitt Partners Torch Insight tool.^31^ We also used data provided by the Health Resources Services Administration to create a variable indicating whether the local system included at least one hospital participating in the 340B program as a covered entity in 2018.^32^

We report descriptive results on the levels and patterns of physician integration with systems by local health system types. Recognizing that many health system characteristics are correlated with each other and levels of integration, we used linear regression to estimate the associations between the measures of integration and each local system characteristic, while controlling for the other local system characteristics. In addition, because larger health systems might be more likely to include a broader range of physician specialties than smaller systems simply due to their size, and are more likely to have certain local system characteristics (eg, a hospital participating in an ACO or the 340B program), we also included local system size (measured by the number of beds in the local system) in the regressions. We also report unadjusted results in the Appendix S1.

## 3 RESULTS

### 3.1 Market‐level results

**FIGURE 1:**
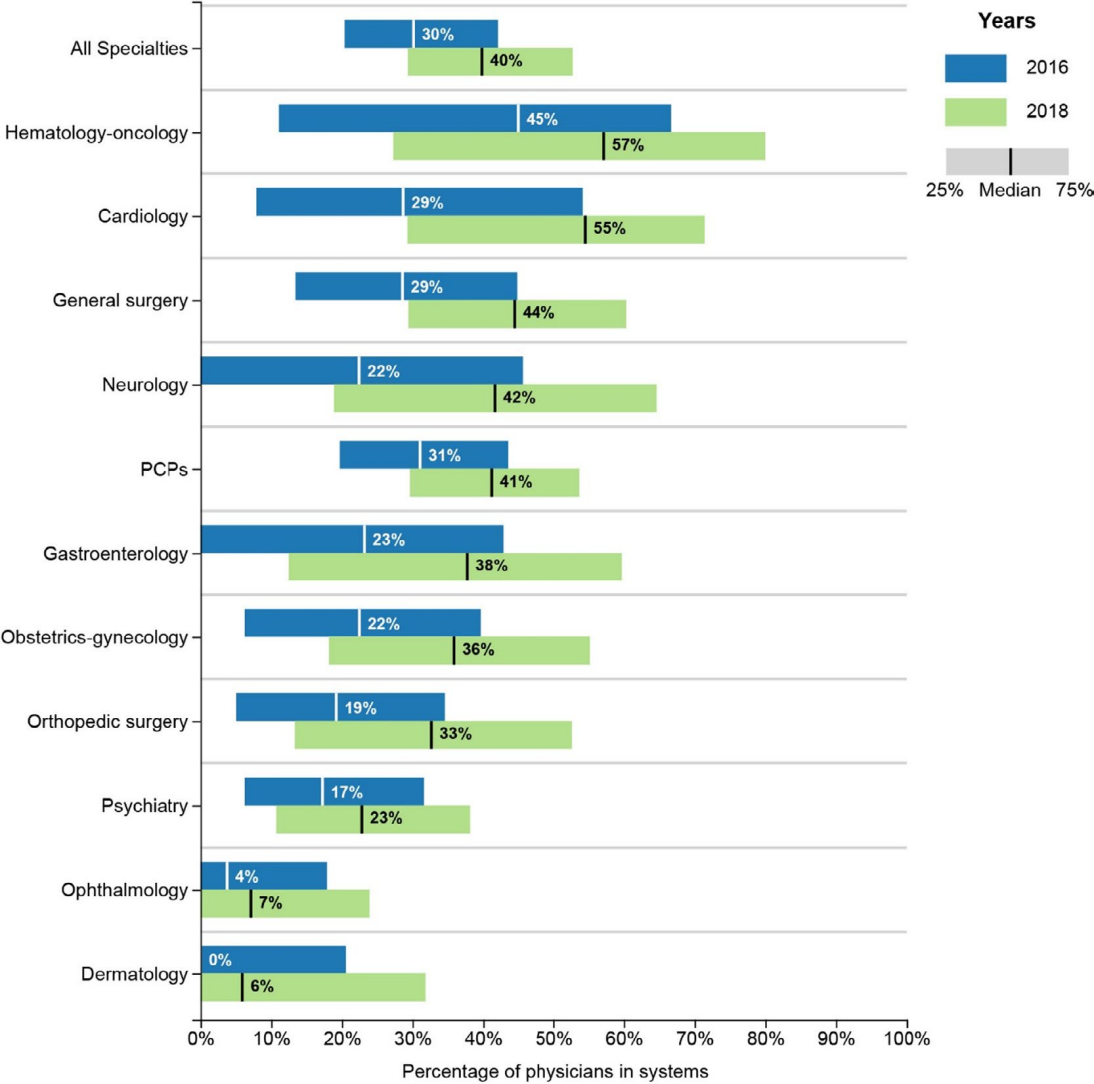
Percentage of physician specialties integrated with systems across MSAs, 2016‐2018. Figure 1 is a boxplot showing the median and interquartile range of the percentage of physician specialties integrated with systems across MSAs in 2016 and 2018 [Color figure can be viewed at wileyonlinelibrary.com]

Across MSAs, the median percentage of physicians integrated with systems increased substantially from 2016 to 2018, from 30% to 40% (Figure 1). The percentage of physicians aligned with systems varied considerably across specialties. In 2018, physicians in specialties whose patients often require inpatient or outpatient hospital services were more likely to be integrated with systems. Hematology‐oncology (57%), cardiology (55%), general surgery (44%), and neurology (42%) were all considerably more likely to be integrated with systems compared to specialists that seldom hospitalize patients, such as ophthalmologists (7%) and dermatologists (6%).

Between 2016 and 2018, system participation increased for primary care and the 10 other physician specialties we examined. Cardiology had the largest percentage point increase in the median value across MSAs: 29% to 55%. Ophthalmology had the smallest percentage point increase: 4% to 7%. In addition, the percentage of physicians integrated with health systems across all specialties varied substantially at the market level. Four of the 10 specialties had an interquartile range (IQR) of greater than 40 percentage points across MSAs (hematology‐oncology, neurology, gastroenterology, and cardiology). Hematology‐oncology had the largest IQR, 25% to 80%.

Table 1 shows the average percentage of physicians that are integrated with systems across markets in 2018 controlling for market population, stratified by whether the market was in the highest or lowest tercile for hospital‐system and insurer concentration. Integration of physicians overall was higher in less concentrated hospital‐system and insurance markets (average values were 7 and 6 percentage points higher, respectively). These associations were largely independent of one another. Hospital‐system and insurance concentration across markets were not highly correlated (*r* = .2).

**TABLE 1:** Mean percentage of physicians integrated with health systems, by physician specialty and measures of market concentration adjusted for MSA population (2018)

Physician specialty	Hospital‐system market HHI		Insurance market HHI	
	Bottom tercile (low concentration)	Top tercile (high concentration)	Bottom tercile (low concentration)	Top tercile (high concentration)
All physicians	45%	38%	43%	37%
Hematology‐oncology	55%	60%	56%	48%
Cardiology	51%	52%	54%	43%
General surgery	49%	42%	45%	43%
Neurology	48%	39%	46%	37%
PCPs	45%	38%	42%	37%
Gastroenterology	42%	36%	40%	34%
Obstetrics‐gynecology	42%	32%	40%	32%
Orthopedic surgery	41%	31%	39%	30%
Psychiatry	28%	25%	27%	21%
Dermatology	24%	15%	20%	14%
Ophthalmology	23%	10%	17%	13%
Average market concentration				
Hospital‐system HHI	3021	9306	5191	6673
Insurance HHI	3075	3797	2208	5092
Number of markets	127	125	127	127

Not<del author="Rachel M Machta" command="Delete" timestamp="1603730378814" title="Deleted by Rachel M Machta on 10/26/2020, 9:39:38 AM" class="reU3">e</del>

Data for one metropolitan statistical area is missing from the measures of hospital‐system and insurance market concentration: The Villages, FL for the hospital‐system measure (due to no general acute care hospitals in that market) and Jacksonville, NC for the insurance market measure (which was not reported in the American Medical Association report). We classified markets as having relatively low concentration (more competitive) if they were in the lowest tercile across markets: HHIs less than or equal to 4696 for hospital‐system concentration and 2696 for insurer concentration. We classified markets as having relatively high concentration (less competitive) if they were in the highest tercile: HHIs greater or equal to 6886 for hospital concentration and 3780 for insurer concentration. We tested whether the differences between groups reported in the figure are statistically significant at the 0.05 level after adjusting for MSA population (for example, statistical significance of differences between markets in the highest and lowest terciles). For hospital‐system HHI, all findings were statistically significant except hematology‐oncology, cardiology, gastroenterology, and psychiatry. For insurance market HHI, all findings were statistically significant except general surgery, gastroenterology, and ophthalmology.

Abbreviations: HHI, Herfindahl–Hirschman Index; PCP, Primary care physician.

There was a consistent pattern of higher physician integration in less concentrated markets across nearly all physician specialties. The exceptions were physicians in two specialties associated with high‐margin hospital services, cardiology and hematology‐oncology, which were slightly more likely to be integrated with systems in markets with higher hospital‐system concentration.

### 3.2 Local health system results

**FIGURE 2:**
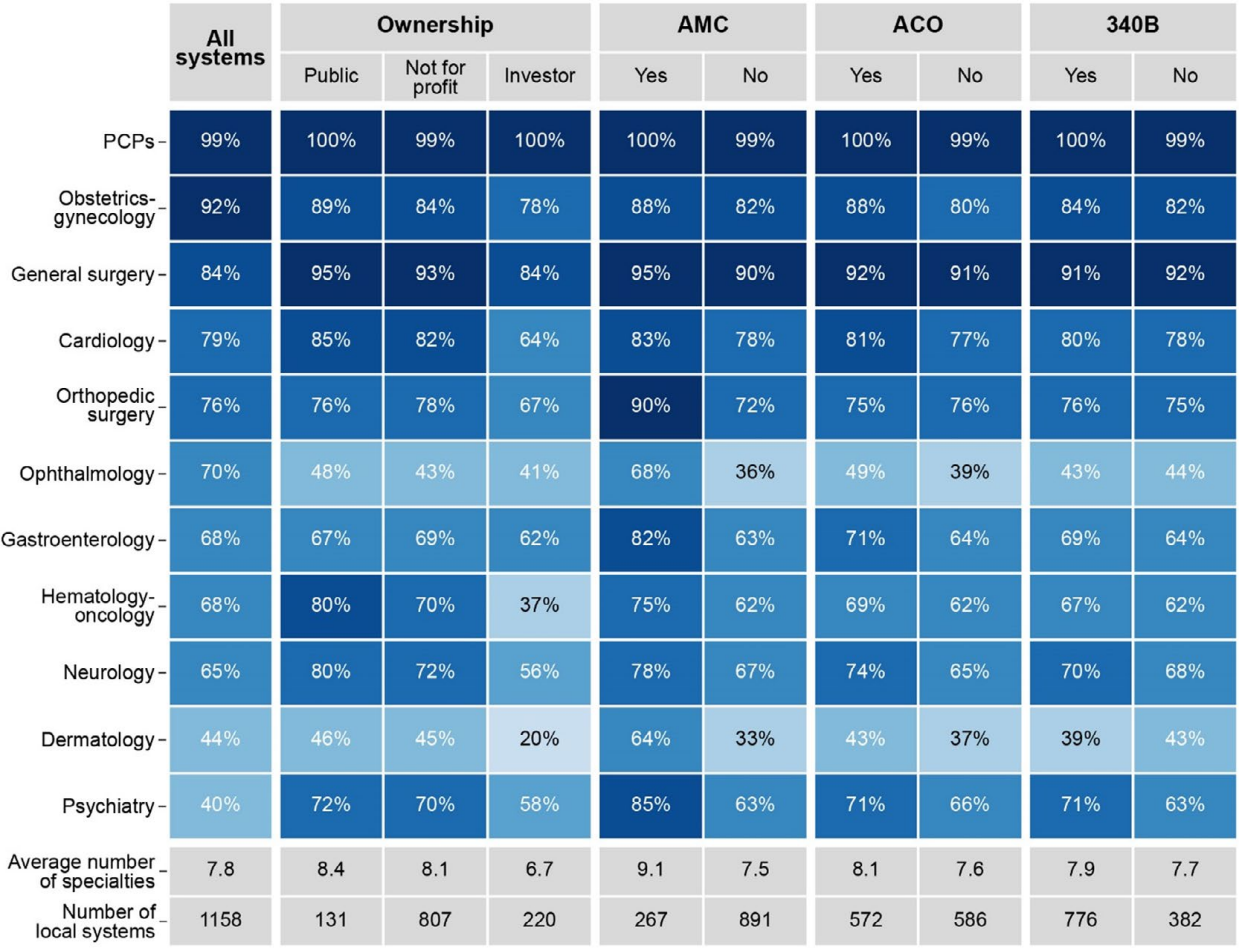
Percentage of systems with physician specialties, by local health system type adjusted by size and local health system characteristics (2018). Figure 2 is a heatmap showing the percentage of local health systems with at least one physician in the market by physician specialty, adjusted by system size and local health system characteristics. Darker colors represent a larger percentage of local systems with at least one of the physician specialties; colors are grouped by 10 percentage points (eg, 0 to 10 percent is one share of blue, 11 to 20 percent is a darker shade, and so on). In the final two rows, it reports the average number of physician specialties by system type (adjusted for system size) and the number of local systems. We tested whether the differences between groups reported in the figure are statistically significant at the 0.05 level (eg, between systems with an AMC and those without an AMC). Putting PCPs aside, all results were statistically significant for investor‐owned systems (compared to public systems) except for orthopedic surgery, gastroenterology, and ophthalmology and for AMCs except for cardiology. Differences were statistically significant for systems participating in an ACO (except for general surgery, cardiology, orthopedic surgery, and psychiatry). The only statistically significant differences between public and not‐for‐profit systems were for hematology‐oncology and neurology and for 340B program, psychiatry. Differences in the average number of specialists were statistically significant for investor‐owned systems (compared to public systems), AMCs, and ACOs. Abbreviations: AMC, Academic Medical Center; ACO, Accountable Care Organization; 340B, Hospital that participates in the 340B program [Color figure can be viewed at wileyonlinelibrary.com]

**FIGURE 3:**
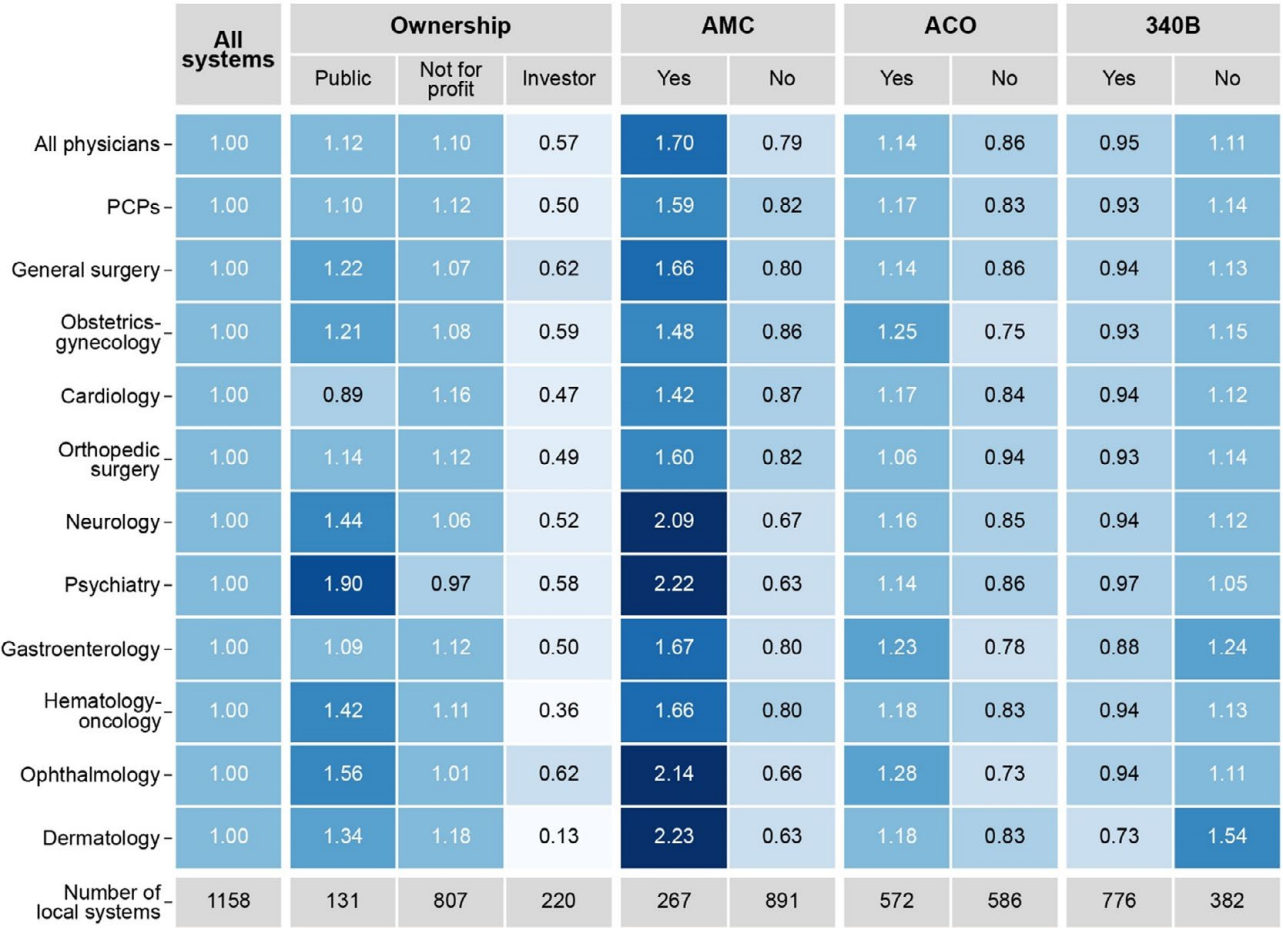
Vertical integration scale, by local health system type, adjusted by size and local health system characteristics. Figure 3 is a heatmap showing the vertical integration scale by health system type adjusted by system size and local health system characteristics. We constructed the vertical integration scale as the number of physicians in the local system divided by the number of beds in the local system and normalized by its mean across all local systems (within physician specialty). Values above one (indicated with darker colors) show integration with physicians above the national average for that specialty. We tested whether the differences between groups reported in the figure are statistically significant at the 0.05 level (eg, between systems with an AMC and those without an AMC). All results were statistically significant for investor‐owned systems (compared to public systems) and for AMCs. Differences were statistically significant for systems participating in an ACO (except orthopedic surgery and dermatology). The only statistically significant differences between public and not‐for‐profit systems were for hematology‐oncology, cardiology, neurology, psychiatry, and ophthalmology and for 340B program, all physicians, PCPs, obstetrics and gynecology, gastroenterology, and dermatology. Abbreviations: AMC, Academic Medical Center; ACO, Accountable Care Organization; PCP: Primary care physicians; 340B, Hospital that participates in the 340B program [Color figure can be viewed at wileyonlinelibrary.com]

Compared to other ownership types, public systems integrated with a broader range of physician specialties. Public systems integrated with physicians in 8.4 specialties on average out of our list of 11, whereas investor‐owned systems integrated with physicians in 6.7 specialties; not‐for‐profit systems were intermediate between these two (Figure 2). Public systems also had more physicians relative to system size than the average system (1.12 as measured by our vertical integration scale), whereas investor‐owned systems had a lower degree of integration (0.57) (Figure 3). For nearly all specialties, public systems were more likely to integrate with physicians, particularly hematology‐oncology and neurology (Figures 2 and 3).

Local systems with an AMC included the highest average number of specialties among the system types we examined (9.1 out of 11 versus 7.5 for systems without AMCs) (Figure 2). Of note, these local systems are much more likely to have at least one physician in the least commonly integrated specialties of ophthalmology and dermatology. As measured by our vertical integration scale, local systems with AMCs also had more physicians relative to local system size, both overall and in each specialty (Figure 3).

Local systems that participate in ACOs were somewhat more likely than local systems that did not participate in ACOs to include at least one physician in specialties relevant to management of prevalent chronic conditions (such as cardiology [81%] and gastroenterology [71%]), and specialties that address common health concerns (such as obstetrics‐gynecology [88%] and ophthalmology [49%]) (Figure 2). Similarly, local systems that participate in ACOs had more integrated physicians overall and for all specialties than local systems that did not participate in ACOs (Figure 3).

Controlling for other system characteristics, local systems that include at least one hospital that participate in the 340B program were slightly more likely than local system that do not include any 340B hospitals to integrate with hematologist‐oncologists (67% vs 62%) and psychiatrists (71% vs 63%), specialties that frequently prescribe expensive drugs for patients (Figure 2).

## 4 DISCUSSION

Given concerns regarding the cost and quality implications of the growth in vertically integrated health systems, we explored patterns of physician integration across markets and health system types to better understand potential motivations driving physician integration. Our research confirms that there are diverse patterns of vertical integration across markets, physician specialties, and health systems.

As expected, specialties that rarely refer patients for hospital services were less likely to be integrated with systems. Although many specialties that frequently refer patients for hospital services have a relatively high degree of system participation, this varies substantially across markets (eg, with an IQR of 25% to 80% for hematology‐oncology) and across specialties (median of 55% for cardiology but 33% for orthopedic surgery). This variation suggests market and system factors play important roles in the likelihood of specialty physician integration in systems.

We also found that the market concentration in both hospital and insurance markets is associated with physician integration across specialties. Low market consolidation by insurers is associated with higher rates of physician integration across specialties, suggesting either hospitals or physician practices (or both) see advantages to integration when there are multiple health plans with whom to negotiate. High hospital‐system consolidation in the market was associated with distinctly lower rates of integration for most physician specialties; but cardiology and hematology‐oncology were exceptions. Integration of these specialties may be so beneficial to systems that local market factors play a more limited role in their strategy.

Local health system ownership was associated with patterns of physician integration. Most notably, investor‐owned systems demonstrate more limited integration with physicians. It may be that in some markets, these systems are more focused on hospital management than on physician integration. Like most systems, the specialty physician affiliation achieved by investor‐owned systems is oriented toward physicians who refer patients for hospital services and thus might offer a timelier return on investment.

Of course, health systems often serve missions beyond ensuring lucrative referrals to their hospital affiliates. Our findings confirm that systems serving distinct missions, like publicly owned systems or systems with AMCs, are more likely to affiliate with specialties unrelated to the physicians’ potential contribution to hospital revenue. Indeed, local systems with AMCs are affiliated with an even broader set of physician specialties (adjusted for system size and local system characteristics) than public systems. This finding suggests that the training and/or research missions of local systems with AMCs may demand a particularly broad array of specialty physicians.^30^

The findings for systems with hospitals that participate in the 340B payment program were not conclusive. Hematologists‐oncologists were somewhat more likely to be affiliated with systems with 340B hospitals, as might be anticipated given their relatively high proportion of revenue attributable to parenteral drugs.^25^ However, whlie psychiatrists (who use expensive prescription drugs in ambulatory care of severe chronic psychiatric conditions) were more likely to be affiliated than hematologists‐oncologists, other specialties associated with high use of infusion therapy were not more likely to be integrated (eg, ophthalmology). Further research will be required to understand how participation in the 340B program may relate to other local system characteristics and the prescribing patterns of the specialties with which they integrate.

There are several limitations to our study. First, while our data includes most systems and physicians, small systems that do not meet the Compendium definition of a health system (eg, those with fewer than 50 physicians nationally) and physicians in rural areas (roughly 4% of physicians [Table S1]) were omitted. In addition, there is no ideal way to define local healthcare markets. For instance, some large urbanized areas include adjacent MSAs. If patients routinely travel to the adjacent MSA for care, these MSAs will be less well suited as proxies for healthcare markets. Third, our analysis is descriptive and cannot be used to infer causality. For example, it could be that systems integrated with certain physician specialties are more likely to participate in ACOs; or conversely, it could be that over time, participation in ACOs serves as motivation for systems to integrate with certain physician specialties. Furthermore, individual associations could be confounded by unmeasured market and system characteristics. We lack the detailed data needed to capture the wide range of strategies and motivations to integrate from the system and physicians’ perspectives that would enable us to build a full model of physician integration and test causal relationships.

Our findings support the need to consider physician‐system integration within the context of the markets in which they operate and for careful local scrutiny of the purposes, risks, and benefits to the local community of such integration. The findings also support consideration of how payment policies could provide incentives to systems to integrate with physician specialties in a way that promotes higher value care. Future research can build off these findings to identify causal relationships between health system types and physician integration strategies, to explore the extent to which systems vary their strategies across markets, and to examine how relationships differ by reliance on traditional fee‐for‐service versus alternative payment models. Finally, it would be helpful to conduct a more thorough exploration of the intersection between competition in healthcare markets and the local health system's competitive position in the market to understand the drivers of physician consolidation across specialties.

## Supporting information

Author matrix

Appendix S1
