# Supplementary material for: Health system integration with physician specialties varies across markets and system types
Source: Health Serv Res. 2020 Dec 7;55(Suppl 3):1062–72. doi: 10.1111/1475-6773.13584 (PMC7720709; doi:10.1111/1475-6773.13584)
Supplement: Supplementary file 2 — Appendix S1 [file HESR-55-1062-s002.docx]

The HCOS and IQVIA OneKey data include most health systems identified in the Compendium (633 out of the 637 in 2018; and 602 out of 625 systems in 2016); the remainder were identified using other data sources (SK&A and the American Hospital Association survey data).

The AHRQ CHSP technical documentation (available at: <https://www.ahrq.gov/chsp/data-resources/compendium/technical-documentation.html>) provides details regarding how health systems were defined and how many systems were excluded to match AHRQ’s Compendium working definition of a health system: at least one general acute care hospital and 50 or more total physicians, including 10 or more total physician’s. In 2018, we started with a list of 1,007 health systems. We then removed 140 systems that lacked at least one non-Federal general acute care hospitals, 214 systems that did not have at least 50 physicians and 5 that lacked at least 10 primary care physicians. In 2016, we started with 1,069 health systems. We then removed 165 systems that lacked at least one non-Federal general acute care hospital, 265 systems that did not have at least 50 physicians and 8 that lacked at least 10 primary care physicians.

OneKey identifies physician affiliations via web searches, telephone verification, and information received from the American Medical Association, and National Plan and Provider Enumeration System, among other sources. Providers are periodically verified via telephone. Each time a medical group practice is verified, so too are the providers within that group. By this means, OneKey can identify inactive physicians, which are excluded from the analysis.

In appendix Table 1, we provide the number of physicians in the IQVIA data by specialty for 2016 (HCOS) and 2018 (OneKey). For comparison, we included the number of physicians in the Association of American Medical Colleges (AAMC) workforce data (available here: <https://www.aamc.org/data-reports/workforce/interactive-data/active-physicians-largest-specialties-2017>). The remainder of this appendix provides unadjusted results for the results provided in the main paper.

**Appendix Table 1. Physicians included in the study**

|  | 2016 | | | 2018 | | | Number of physicians in AAMC involved in patient care in 2017 |
| --- | --- | --- | --- | --- | --- | --- | --- |
|  | Number of physicians in HCOS | Percentage in MSAs | Percentage in MSA and systems | Number of physicians in OneKey | Percentage in MSAs | Percentage in MSA and systems |  |
| **All physician specialties** | 911,971 | 96 | 37 | 987,147 | 96 | 49 | 892,856 |
| Hematology-oncology | 9,698 | 96 | 49 | 11,131 | 97 | 60 | 15,410 |
| Cardiology | 25,212 | 97 | 39 | 25,972 | 97 | 57 | 22,211 |
| Neurology | 15,091 | 97 | 37 | 16,409 | 97 | 54 | 13,717 |
| General surgery | 30,969 | 93 | 36 | 33,604 | 94 | 50 | 25,042 |
| PCPs | 319,702 | 94 | 36 | 335,720 | 95 | 47 | 346,495 |
| Obstetrics-gynecology | 42,615 | 95 | 31 | 44,676 | 95 | 45 | 41,656 |
| Gastroenterology | 15,141 | 97 | 32 | 16,548 | 97 | 46 | 14,747 |
| Orthopedic surgery | 24,201 | 94 | 28 | 25,345 | 94 | 42 | 19,001 |
| Psychiatry | 34,568 | 96 | 24 | 37,075 | 96 | 31 | 38,205 |
| Dermatology | 12,182 | 97 | 21 | 13,622 | 97 | 30 | 12,051 |
| Ophthalmology | 20,357 | 96 | 19 | 21,361 | 96 | 30 | 18,817 |

Abbreviations: PCP: Primary care physicians; AAMC: Association of American Medical Colleges; MSA: Metropolitan Statistical Area

**Appendix Table 2. Mean percentage of physicians integrated with health systems, by physician specialty and measures of market concentration, unadjusted (2018).**

|  | **Hospital-system market HHI** | | **Insurance market HHI** | |
| --- | --- | --- | --- | --- |
| **Physician specialty** | **Bottom tercile**  **(low concentration)** | **Top tercile**  **(high concentration)** | **Bottom tercile**  **(low concentration)** | **Top tercile**  **(high concentration)** |
| All physicians | 45% | 38% | 43% | 37% |
| Hematology-oncology | 55% | 60% | 56% | 48% |
| Cardiology | 51% | 52% | 54% | 43% |
| General surgery | 49% | 42% | 46% | 43% |
| Neurology | 48% | 39% | 46% | 37% |
| PCPs | 45% | 38% | 43% | 37% |
| Gastroenterology | 42% | 36% | 40% | 34% |
| Obstetrics-gynecology | 43% | 32% | 40% | 32% |
| Orthopedic surgery | 40% | 31% | 39% | 29% |
| Psychiatry | 29% | 25% | 27% | 21% |
| Dermatology | 24% | 15% | 21% | 13% |
| Ophthalmology | 24% | 9% | 18% | 13% |
| **Average market concentration** |  |  |  |  |
| Hospital-system HHI | 3021 | 9306 | 5191 | 6673 |
| Insurance HHI | 3075 | 3797 | 2208 | 5092 |
| **Number of markets** | 127 | 125 | 127 | 127 |

Abbreviations: HHI, Herfindahl–Hirschman Index; PCP, Primary care physician

Data for one metropolitan statistical area is missing from the measures of hospital-system and insurance market concentration: The Villages, FL for the hospital-system measure (due to no general acute care hospitals in that market) and Jacksonville, NC for the insurance-market measure (which was not reported in the American Medical Association report). We classified markets as having relatively low concentration (more competitive) if they were in the lowest tercile across markets: HHIs less than or equal to 4,696 for hospital-system concentration and 2,696 for insurer concentration. We classified markets as having relatively high concentration (less competitive) if they were in the highest tercile: HHIs greater or equal to 6,886 for hospital concentration and 3,780 for insurer concentration.

**Appendix Figure 1.** **Percentage of systems with physician specialties, by local health system type, unadjusted (2018)**


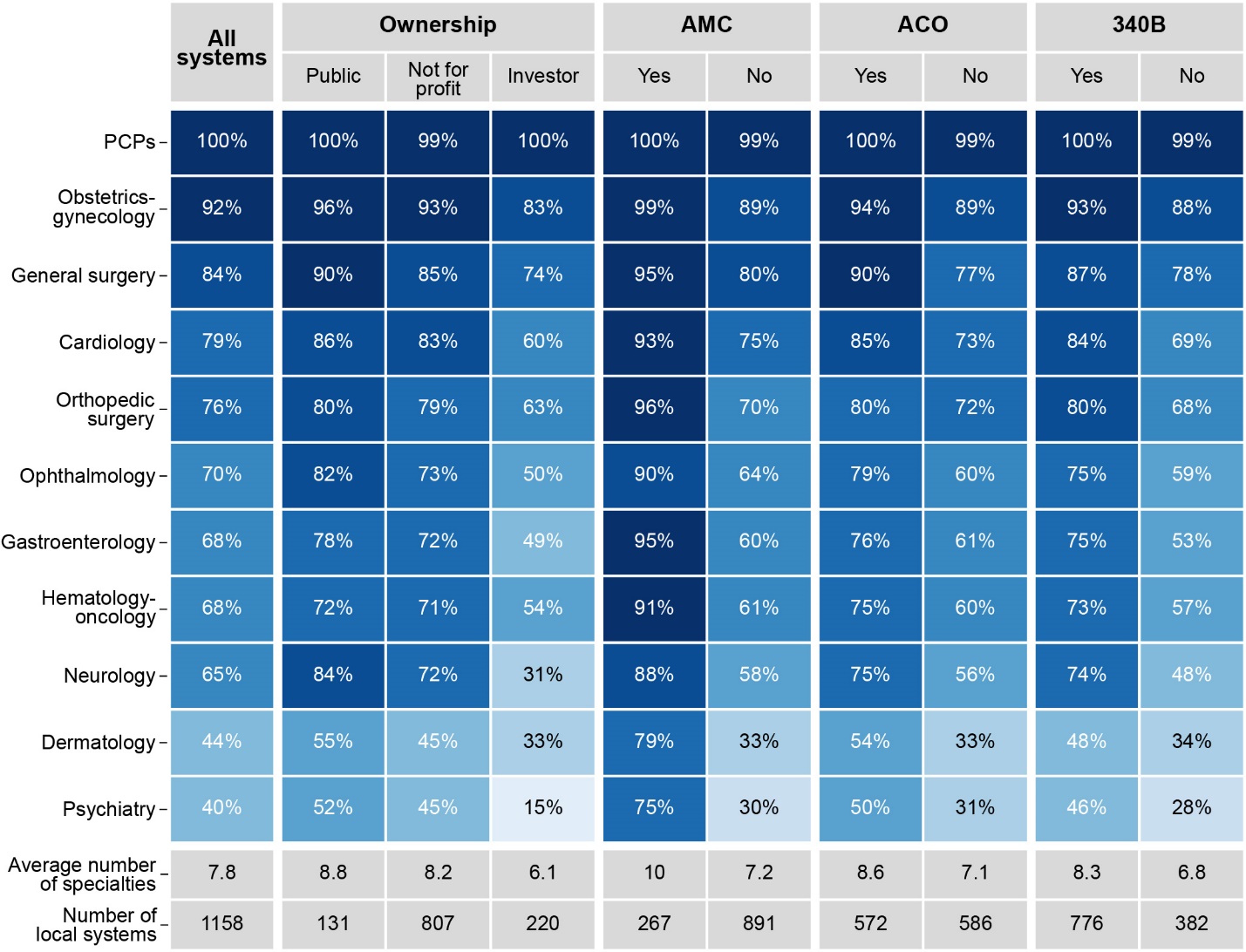


Appendix Figure 1 is a heatmap showing the percentage of local health systems with at least one physician in the market by physician specialty. Darker colors represent a larger percentage of local systems with at least one of the physician specialties; colors are grouped by 10 percentage points (e.g., 0 to 10 percent is one share of blue, 11 to 20 percent is a darker shade, and so on). In the final two rows, it reports the average number of physician specialties by system type (adjusted for system size) and the number of local systems.

Abbreviations: AMC, Academic Medical Center; ACO, Accountable Care Organization; 340B, Hospital that participates in the 340B program.

**Appendix Figure 2. Vertical integration scale, by local health system type, unadjusted (2018)**


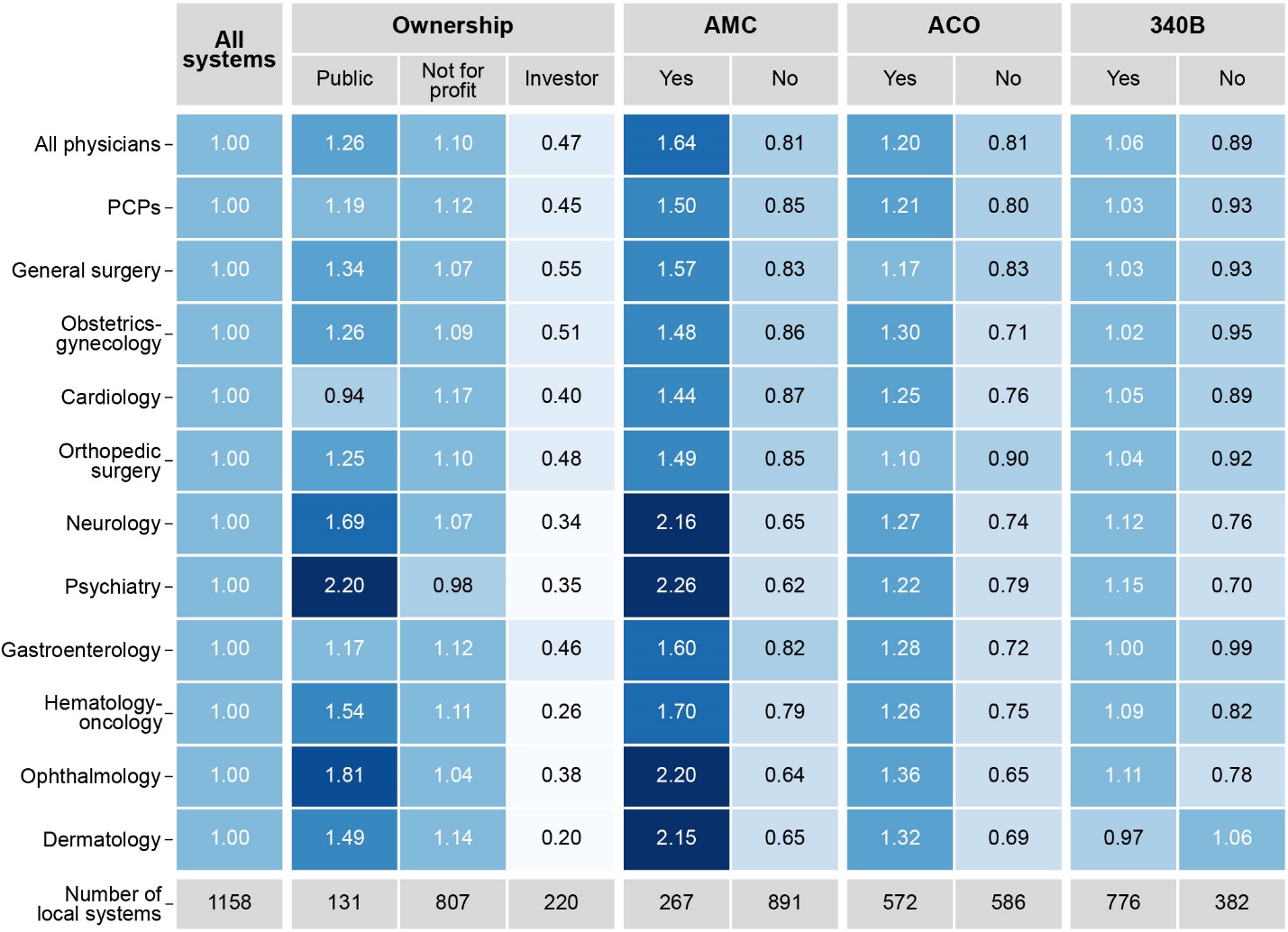


Appendix Figure 2 is a heatmap showing our vertical integration scale by health system type. We constructed the vertical integration scale as the number of physicians in the local system divided by the number of beds in the local system and normalized by its mean across all local systems (within physician specialty). Values above one (indicated with darker colors) show integration with physicians above the national average for that specialty.

Abbreviations: AMC, Academic Medical Center; ACO, Accountable Care Organization; PCP: Primary care physicians; 340B, Hospital that participates in the 340B program.
